# Effect of Percutaneous Kyphoplasty on the progression of intervertebral disc degeneration: a retrospective cohort study

**DOI:** 10.1186/s13018-023-03627-6

**Published:** 2023-03-06

**Authors:** Tiemure Wu, Xiao Han, Wei Tian, Lifang Wang, Chao Wang

**Affiliations:** 1 Department of Spine Surgery, Beijing Jishuitan Hospital, Fourth Clinical College of Peking University, Beijing, 100035 China; 2 Department of Statistics, Beijing Jishuitan Hospital, Fourth Clinical College of Peking University, Beijing, 100035 China; 3 Beijing Research Institute of Traumatology and Orthopaedics, Beijing, China

**Keywords:** Percutaneous Kyphoplasty, Cement leak, Polymethylmethacrylate, Intervertebral disc degeneration

## Abstract

**Background:**

The effect of percutaneous kyphoplasty (PKP) or rather polymethylmethacrylate (PMMA) on adjacent intervertebral discs is still controversial. The evidence from experimental study to clinical study presents bipolar conclusions. In this study, we investigated the effect of PKP on adjacent intervertebral disc degeneration (IDD).

**Methods:**

The experimental group included adjacent intervertebral discs of vertebrae treated with the PKP procedure, and the control group included adjacent intervertebral discs of non-traumatized vertebrae. All measurements were taken by magnetic resonance imaging or X-ray. The intervertebral disc height, the modified Pfirrmann grading system (MPGS), and its differences with Klezl Z and Patel S (ZK and SP) classifications were compared.

**Results:**

A total of 264 intervertebral discs from 66 individuals were selected for the study. The comparison of intervertebral disc height between the two groups pre and post-operatively resulted in a *p*-value of > 0.05. No significant change was observed in the adjacent discs in the control groups post-operatively. Post-operatively, the mean Ridit increased significantly from 0.413 to 0.587 in the upper disc and from 0.404 to 0.595 in the lower disc in the experimental group. The comparison of MPGS differences showed that the predominant value was 0 in the Low-grade leaks group and 1 in the Medium and high-grade leaks group.

**Conclusions:**

The PKP procedure can accelerate adjacent IDD, but it does not cause disc height changes in the early stage. The quantity of cement leaking into the disc space positively correlated with the rate of disc degeneration progression.

## Background

Osteoporotic vertebral compression fractures (OVCFs) are becoming increasingly common worldwide owing to the ageing of the population [1–3]. Kyphoplasty is a widely recognized technique used for this type of fracture because of its minimally invasive nature, rapid pain relief, kyphosis correction, vertebral height recovery, and increased stability.

Currently, polymethylmethacrylate (PMMA) is the most popular and commonly used filler material for vertebral body cement augmentation. Despite the demonstrated benefits, some authors have stated that percutaneous kyphoplasty (PKP) increases the risk for subsequent vertebral fractures [4]. Authors have also proposed different theories to explain this, including adjacent structural degeneration; however, the effect of the cement on the intervertebral discs and adjacent vertebrae remains unclear.

Park CK et al. and Belkoff SM et al. measured the internal vertebral body temperature during the polymerization of cement used for vertebroplasty and recorded high temperatures that would damage the adjacent intervertebral discs and accelerate their degeneration [5, 6]. Nevertheless, Aebli N et al. observed that the temperature did not exceed 47 °C and concluded that PMMA would not cause thermal damage to the intervertebral discs and spinal cord [7].

Another hypothesis is that because of its chemical composition (methyl methacrylate monomer) or contrast agent (barium sulphate), PKP may cause toxic side effects to adjacent intervertebral discs when cement leaks into the adjacent disc space, resulting in degeneration [8–11].

Other theories propose that blockade of the endplate nutritional pathway can interfere with the supply of nutrition and cause intervertebral disc degeneration. However, this hypothesis is poorly supported by animal models [12–14].

Intervertebral disc cement leakage may increase the risk of new adjacent vertebral fractures and accelerate intervertebral disc degeneration [4, 15–17]. However, it is unclear whether these fractures are caused by a change in pressure distribution or osteoporosis evolution. Recently, Pachowsky ML et al. reported increased degeneration of adjacent intervertebral discs following kyphoplasty using quantitative T2 mapping [18]. Luo J et al*.* performed a biomechanical study on 28 cadaveric spines and showed that vertebral fractures had an unfavourable effect on compressive load sharing and increased vertebral deformations at the fracture and adjacent levels. These effects can be partially reversed with vertebroplasty [19].

On account of contradictory literary evidence regarding a link between PKP and subsequent vertebral fractures, the risk of degenerative changes associated with intradiscal cement leakage has not been fully explored, and currently, the evidence in the literature remains limited to animal studies. We also found no retrospective cohort study that investigated the relationship among disc height, disc degeneration (visualized using magnetic resonance imaging [MRI]), and post-PKP cement leakage. Therefore, in this study, we aimed to assess the effects of PKP on adjacent intervertebral discs using MRI and X-ray studies.

## Methods

**Fig. 1:**
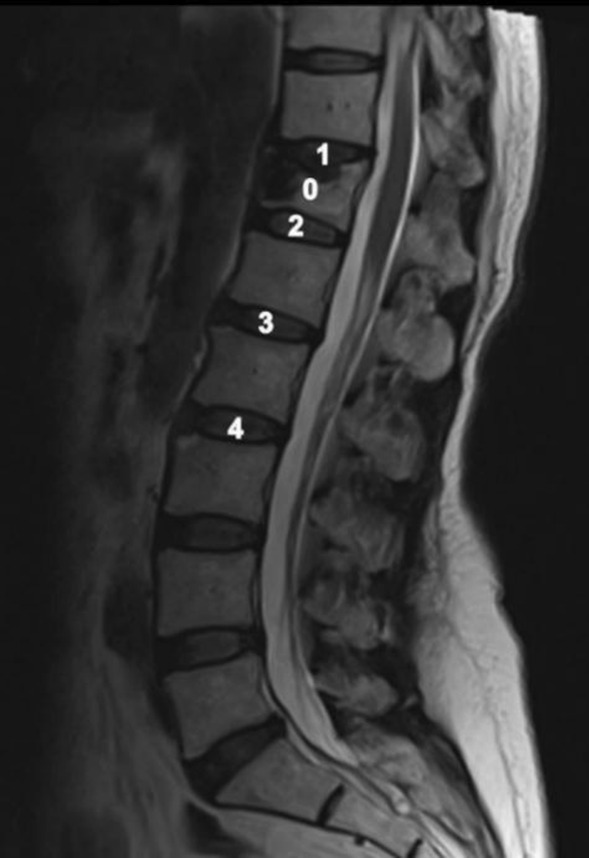
MRI of spine assessments. 0: PKP vertebral. 1. Upper disc of the kyphoplasty vertebral group (KVG). 2. Lower disc of the KVG. 3. Upper disc of the non-traumatized vertebral group (NTVG). 4. Lower disc of the NTVG

We retrospectively reviewed MR and X-ray images of 66 patients who underwent PKP, performed by three surgeons between January 2000 and December 2019. We compared different spinal sections of the same patient to ensure the minimization of factors affecting the results. The adjacent intervertebral discs superior and inferior to the vertebra that underwent the procedure were included in the experimental groups. Intervertebral discs superior and inferior to the nearby non-traumatized control vertebra of the same patient were included in the control group (Fig. 1). The control and experimental vertebrae needed to be in the same segment: the thoracic vertebrae segment (T1–T9), thoracolumbar vertebrae segment (T10–L2), or lower lumbar vertebrae segment (L3–L5). The inclusion criteria were as follows: OVCFs and treatment with PKP. The exclusion criteria were as follows: insufficient preoperative imaging studies (X-ray and MRI); insufficient preoperative follow-up time (< 12 months); multiple vertebral fractures; pathological fractures; bedridden patients; insufficient intervertebral discs on images to be measured; and adjacent intervertebral disc injury caused by OVCFs.

### MRI evaluation

**Fig. 2:**
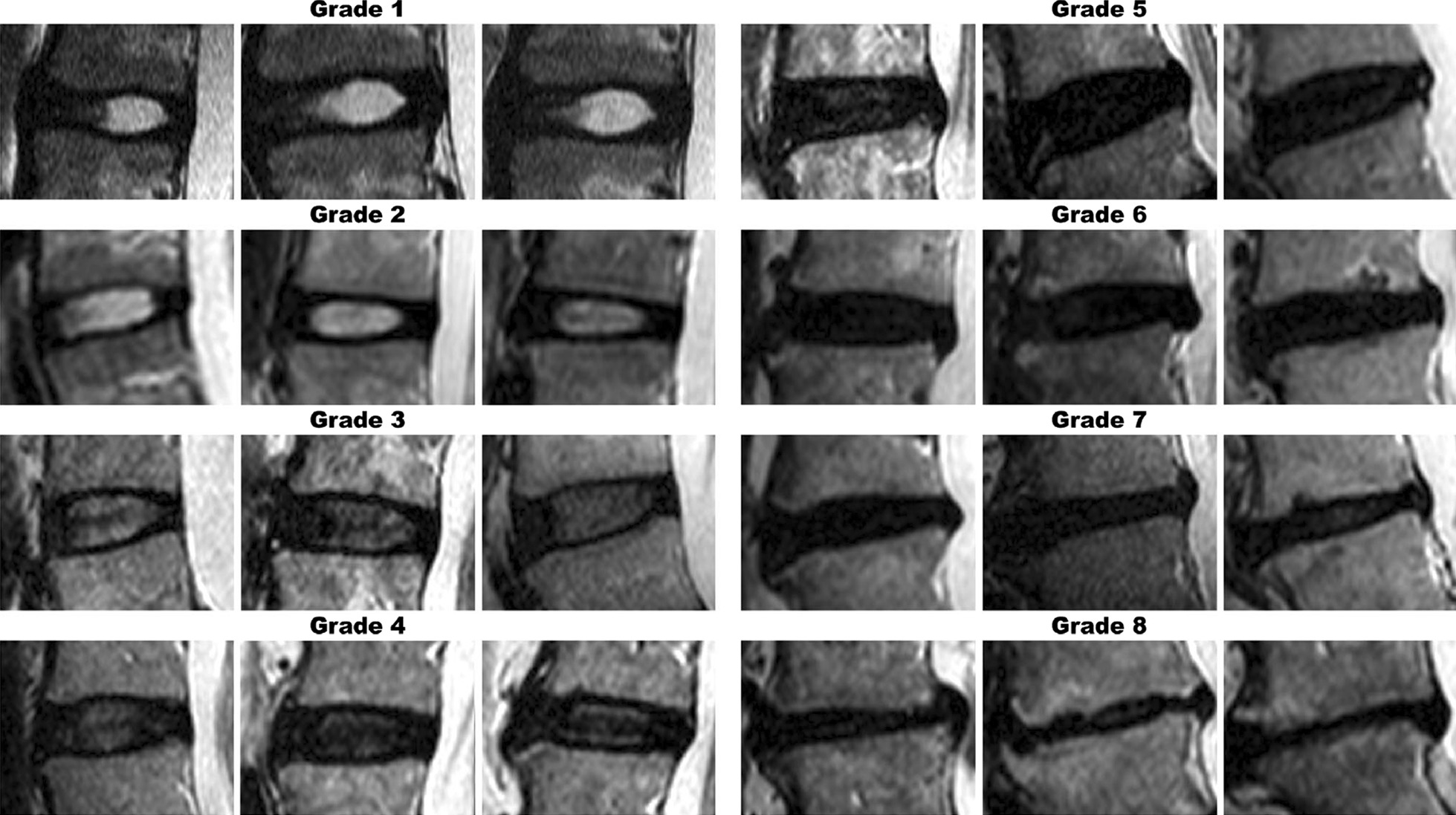
An image reference panel showing the increasing severity of disc degeneration. The pertinent features of each grade are described in Table 1. Three images reflect the inherent variability across each grade. This figure is reproduced from Griffith, J. F. et al*.*^21^

**Fig. 3:**
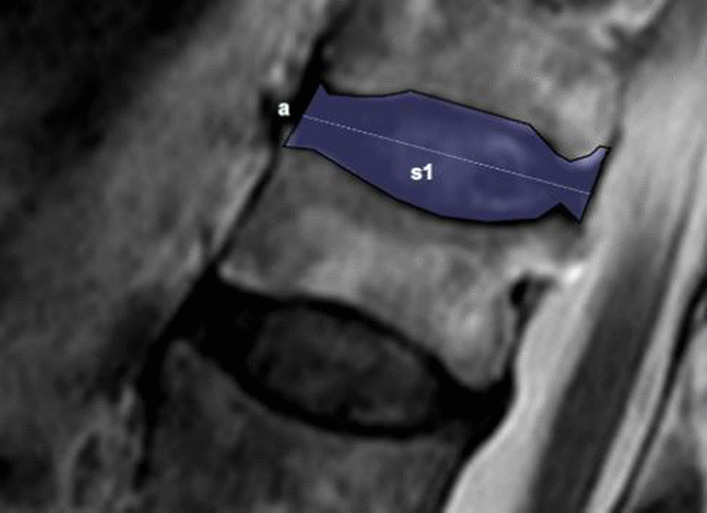
Intervertebral disc height measurement. The height of the disc is approximately equal to the area (S1) divided by the length of the disc (**a**)

Evaluation of T2-weighted MR images was performed using the modified Pfirrmann grading system (MPGS), which comprises eight grades (Fig. 2 and Table 1) [20]. The first MRI must be performed at the time of the fracture just after completing surgery, and the last MRI must be performed at least 1 year after the day of surgery. All studies were performed at Jishuitan Hospital to reduce errors caused by different equipment and measuring software. The disc area was defined by the boundaries of the anterior longitudinal ligament, superior disc-vertebral interface, posterior longitudinal ligament, and inferior disc-vertebral interface with the MATLAB® (Version 9.11, R2021b, The MathWorks, Inc, Natick, USA) freehand region of interest measurement tool. Intervertebral disc height was determined by dividing the disc area by the length of the horizontal line aligned in the anterior and posterior boundaries of the intervertebral disc [21], as shown in Fig. 3.

**Table 1:** Modified grading system for lumbar disc degeneration*

Grade	Signal from the nucleus and inner fibres of the annulus	Distinction between inner and outer fibres of the annulus at the posterior aspect of the disc	Height of disc
1	Uniformly hyperintense, equal to CSF*	Distinct	Normal
2	Hyperintense (> presacral fat and < CSF*) ± hypointense intranuclear cleft	Distinct	Normal
3	Hyperintense though < presacral fat	Distinct	Normal
4	Mildly hyperintense (slightly > outer fibresof the annulus)	Indistinct	Normal
5	Hypointense (= outer fibres of the annulus)	Indistinct	Normal
6	Hypointense	Indistinct	< 30% reduction in disc height
7	Hypointense	Indistinct	30–60% reduction in disc height
8	Hypointense	Indistinct	> 60% reduction in disc height

This table is reproduced from Griffith et al*.* [21]. Different levels are determined by signals from the nucleus and inner fibres of the annulus, distinction between inner and outer fibres of the annulus at the posterior aspect of the disc, and disc height

^*^CSF Cerebrospinal fluid

### X-ray evaluation

**Fig. 4:**
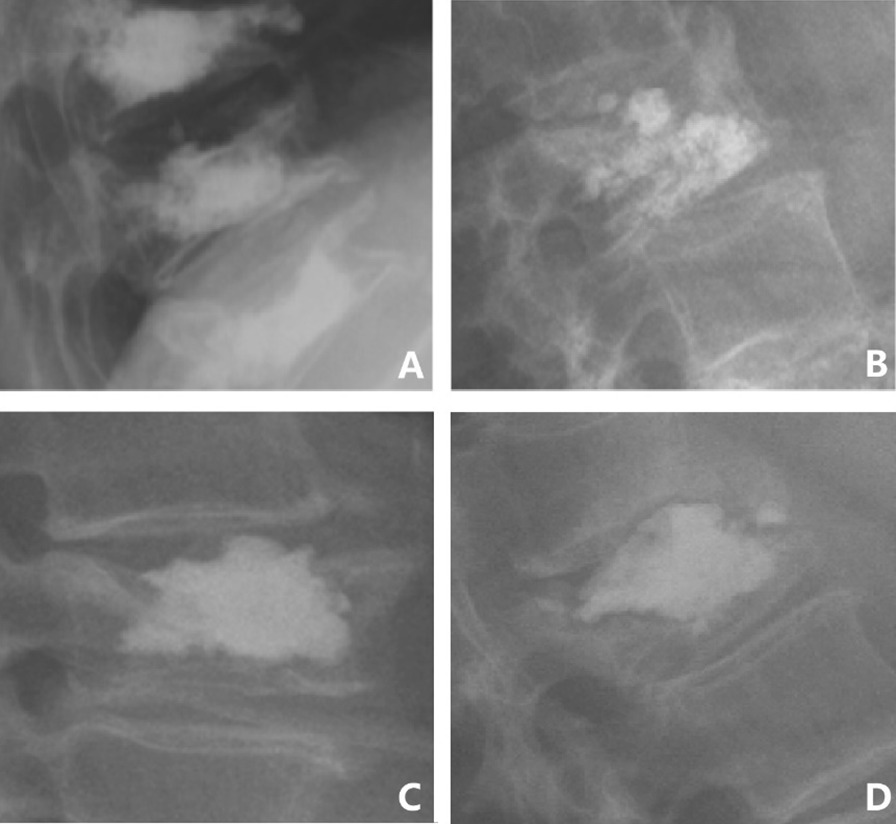
The ZK and SP classification. This figure is reproduced from Jamjoom et al. [22]. The ZK and SP classification system is based on the leakage of cement into the disc space. a: Grade I, cement leakage into the disc space was considered minimal/cloud. b: Grade II, cement leakage filling < 20% of the disc space. c: Grade III, cement leakage filling 20–40% of the disc space. d: Grade IV, cement leakage filling > 40% of the disc space

Radiographic studies were performed on the same date as MRI. The amount of intradiscal cement leakage was quantified using a grading system proposed by two authors, the Klezl Z and Patel S classification (ZK and SP) [22]. In this classification system, grade I indicates that cement leakage into the disc space was minimal/cloud; grade II is when cement leakage fills < 20% of the disc space, grade III is when cement leakage fills 20–40% of the disc space, and grade IV is when cement leakage fills > 40% of the disc space (Fig. 4). In addition, we calculated the disc height correspondence.

### Statistical analysis

Statistical analyses were performed using SPSS for Windows®, Versión 25 (IBM, Armonk, NY, USA.). The experimental and control groups were independent of each other. Disc height was quantitatively measured preoperatively and post-operatively, and the comparison between the two groups was performed using an independent sample t test; the level of significance was set at a *p*-value < 0.05.

We determined the MPGS grades immediately after surgery and during regular follow-up and compared them between the experimental and control groups using the Ridit analysis. Furthermore, based on the quantity of intradiscal cement leaked (ZK and SP classification), patients were divided into two groups for analysis: low-grade leaks (LGL, grade I) and medium- and high-grade leaks (MHGL; grades II–IV). The differences in the MPGS grades were compared between the two groups through Mann–Whitney U analysis to examine the relationship between cement leakage into the disc space and disc degeneration.

## Results

Between January 2012 and December 2019, 264 intervertebral discs from 66 patients were analysed in this study; the median patient age was 74.6 years (range, 57–99 years), and 90% of the patients were women (60). Overall, 18% (12) of the fractures occurred in thoracic vertebrae (T1–T9), 53% (35) in thoracolumbar vertebrae (T10–L2), and 29% (19) in lower lumbar vertebrae (L3–L5). The average re-evaluation time was 927.1 days (514–3060 days).

We compared the pre- and post-operative intervertebral disc heights between the kyphoplasty vertebral group (KVG) and non-traumatized vertebral group (NTVG) (Table 2). There were no significant differences observed in the groups.

**Table 2:** Comparison of pre- and post-operative disc height differences between the experimental and control groups

Groups		Kyphoplasty vertebral group	Non-traumatized vertebral group	*p*
Upper disc	Preoperative	9.9 ± 2.6	9.8 ± 3.3	0.786
	Post-operative	8.5 ± 2.4	8.5 ± 2.6	0.946
Lower disc	Preoperative	11.3 ± 7.5	9.8 ± 2.7	0.125
	Post-operative	9.1 ± 2.8	8.8 ± 3.0	0.527

Comparing the disc height pre- and post-operatively between the control and experimental groups; there is no statistically significant difference. *p*: *p*-value of the independent sample *t* test

**Fig. 5:**
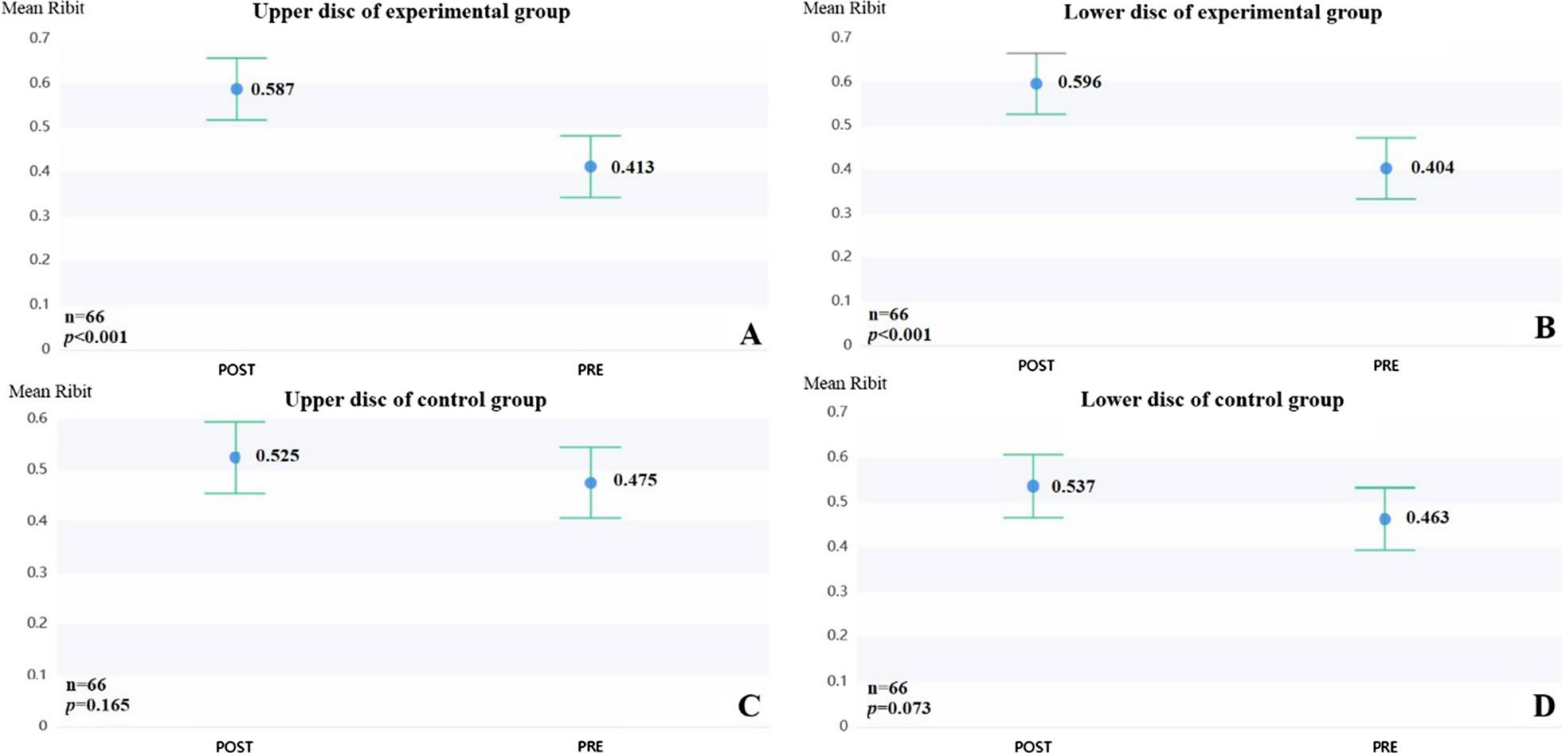
Comparison of pre- and post-operative Modified Pfirrmann Grading System grades between the experimental and control groups (Ridit analysis). Disc degeneration post-operatively was demonstrated in the experimental group, but was not observed in the control group. **A:** Upper disc of the experimental group; **B:** Lower disc of the experimental group; **C:** Upper disc of the control group; **D:** Lower disc of the control group *Pre* Preoperative, *Post* Post-operative

A summary of the comparisons of intervertebral disc degeneration between the two groups using the Modified Pfirrmann Grading System (MPGS) is presented in Table 3 and Fig. 5. To evaluate the advanced degeneration, a Ridit analysis was performed. No significant change was observed in the adjacent discs in the NTVG post-operatively; however, a significant difference was observed in the KVG. In the upper disc group, the mean Ridit increased significantly (*p* < 0.001) from 0.413 to 0.587 post-operatively, which indicates that disc degeneration occurred more quickly in the experimental group. The same phenomenon could be observed in the lower disc group where the mean Ridit increased significantly (*p* < 0.001) from 0.404 to 0.595 post-operatively.

**Table 3:** Comparison of pre- and post-operative Modified Pfirrmann Grading System (MPGS) grades between the experimental and control groups

Groups			Modified Pfirrmann Grading System (%)								Total	Mean Ridit	*p*
			1	2	3	4	5	6	7	8			
KVG	Upper disc	Pre	0	2(3)	20(20.3)	38(57.6)	6(9.1)	0	0	0	66	0.413	< 0.001
		Post	0	0	11(16.7)	27(40.9)	24(36.4)	4(6.1)	0	0	66	0.587	
	Lower disc	Pre	0	1(1.5)	25(37.9)	34(51.5)	6(9.1)	0	0	0	66	0.404	< 0.001
		Post	0	0	0	12(18.2)	26(39.4)	24(36.4)	4(6.1)	0	66	0.596	
NTVG	Upper disc	Pre	0	0	20(30.3)	30(45.5)	15(22.7)	1(1.5)	0	0	66	0.475	0.165
		Post	0	0	16(24.2)	27(40.9)	20(30.3)	3(4.5)	0	0	66	0.525	
	Lower disc	Pre	0	0	26(39.4)	26(39.4)	14(0.2)	0	0	0	66	0.463	0.073
		Post	0	0	20(30.3)	22(33.3)	22(33.3)	1(1.5)	1(1.5)	0	66	0.537	

On comparing the MPGS grades, no statistically significant difference was noted in disc height in either the experimental or control group pre- and post-operatively. *Pre* Preoperative, *Post* Post-operative, *KVG* Kyphoplasty vertebral group. *NTVG* Non-traumatized vertebral group. *p:* p-value of Ridit analysis

### Relationship between ZK and SP grade and MPGS differences

The instances of cement leakage into the 66-disc spaces were graded as follows: grade I, 19 (28.8%); grade II, 23 (34.8%); grade III, 17 (25.8%); and grade IV, 7 (10.6%), as shown in Table 4.

**Table 4:** Findings of 66 episodes of intradiscal cement leak according to the leak severity

Feature	ZK and SP grade							
Grade	I		II		III		IV	
Number/proportion	*n*	%	*n*	%	*n*	%	*n*	%
Values	19	28.8	23	34.8	17	25.8	7	10.6

The comparison of the cement leak disc MPGS differences between the two groups is presented in Table 5, The comparison of MPGS differences between the LGL group and the MHGL group showed statistically significant differences (0.017): the predominant value was 0 in the LGL group (68.4%) and 1 in the MHGL group (53.2%). This means that medium- and high-grade leaks cause more disc degeneration than low-grade leaks.

**Table 5:** Comparing the cement leak disc MPGS differences between the two groups of ZK and SP grade in the experimental group

ZK and SP grade	MPGS differences (%)				*p*
	0	1	2	3	
LGL	13 (68.4)	4 (21.1)	2 (10.5)	0 (0.0)	0.017
MHGL	15 (31.9)	25 (53.2)	5 (10.6)	2 (4.3)	

ZK and SP grades were positively related to the modified Pfirrmann grading system (MPGS), *LGL* Low-grade leaks, grade I; *MHGL* Medium- and high-grade leaks, grades II–IV, *p*: *p*-value of the Mann–Whitney U test

## Discussion

OVCFs appear to have an increasing incidence rate because of the ageing population. Conservative treatment is effective in the management of vertebral body fractures without neurological impairment [23]. However, painful osteoporotic vertebral fractures or spinal instability often necessitate surgical intervention. Evidence suggests that PKP is safe and accelerates recovery in the treatment of OVCFs [24].

Since Galibert P et al. investigated the intravertebral injection of PMMA in patients with vertebral haemangiomas [25], numerous studies have reported the effect of PKP and PMMA on vertebral bodies or intervertebral discs, but the results are variable [26–30]. Therefore, a comprehensive study of degeneration is required to investigate the relationship between changes in disc height, MRI visualization of disc degeneration, and effect of cement leakage into the adjacent intervertebral space.

On comparing pre- and post-operative intervertebral disc height differences between the experimental and control groups, all p-values were more than 0.05, indicating that PKP does not lead to significant changes in the height of the intervertebral discs. However, Shen M et al. reported that intradiscal cement leakage can cause intervertebral disc degeneration [31]. Another study by Salamat S et al. [21] reported that a Pfirrmann score of grade 5 is linked to significantly lower quantitative disc height scores. Despite disc degeneration being observed in the final images, the timescale over which disc height reduces is unclear. Verlaan JJ et al. demonstrated no signs of intervertebral disc degeneration in goats after blockage of one adjacent endplate for 6 months [32]. Krebs J et al. discovered that cement augmentation did not aggravate disc degeneration after 12 months of cement augmentation, even when 80% of both endplates of an intervertebral disc were in contact with PMMA [33]. Given these findings, we believe that there would be no obvious disc height reduction in the early stage of evolution.

Overall disc degeneration, represented by increasing MPGS grade, increased more significantly in the experimental group, as shown in Fig. 5 and Table 3. This suggests that PKP use may result in the acceleration of intervertebral disc degeneration; however, evidence from animal models does not corroborate this. Hutton et al. confirmed that a blocked endplate nutritional process with bone cement within a dog model for up to 70 weeks would not cause obvious degeneration within the intervertebral disc [12]. Kang et al. discovered that after 3 months of bone cement intervention, interference in each endplate pathway in an immature porcine model may cause intervertebral disc degeneration (IDD) [34]. Yin S et al. found that the inhibition of each endplate pathway with cement injection may block the endplate nutritional route and that severe endplate nutritional pathway inhibition may result in IDD at 48 weeks [13]. However, in a clinical investigation, Pachowsky ML et al*.* reported that quantitative T2 mapping showed increased degeneration in adjacent intervertebral discs following kyphoplasty, which is consistent with our study [18].

Assuming PMMA can accelerate the degeneration of the intervertebral disc, it is likely that more cement leakage will cause more intervertebral disc degeneration. All the results were statistically significant and consistent with the findings of the study by Jamjoom et al. [22]. The result can be explained by the study findings reported by Kang et al. [34], who found that 48 weeks after blocking the endplate nutritional route, both endplate nutritional pathways showed obvious IDD changes in goats, including significant disc height reduction and higher disc degeneration grading compared with the control group.

This study had a few limitations. First, IDD could be affected by the type of fracture, and endplate damage caused by the fracture could affect the degree of degeneration. Second, we did not observe obvious disc height reduction for a longer period. Thus, long-term studies and detailed analyses of the effect of the endplate assessed using computed tomography are needed.

## Conclusions

Our findings indicate that PKP does not cause changes in disc height between the experimental and control groups in the early stages. Pfirrmann grade changes demonstrate that PKP can accelerate IDD. The quantity of cement leaking into the disc space positively correlates with the rate of disc degeneration progression. Further long-term studies are required to determine the effect of PKP on disc height.

## Data Availability

The dataset supporting the conclusions of this article can be obtained by contacting the corresponding author.

## Abbreviations

IDD: Intervertebral disc degeneration
KVG: Kyphoplasty vertebral group
MPGS: Modified Pfirrmann grading system
MRI: Magnetic resonance imaging
NTVG: Non-traumatized vertebral group
PKP: Percutaneous Kyphoplasty
ZK: Z Klezl
SP: S Patel
LGL: Low-grade leaks
MHGL: Medium and high-grade leaks
